# Neue Forschungsfragen in der Geschichte der deutschsprachigen Sexualwissenschaft und Sexualmedizin

**DOI:** 10.1007/s00120-023-02091-8

**Published:** 2023-07-10

**Authors:** Matthis Krischel, Richard Kühl, Dana Mahr

**Affiliations:** 1 Institut für Geschichte, Theorie und Ethik der Medizin, Centre for Health and Society, Medizinische Fakultät, Heinrich-Heine-Universität Düsseldorf, Moorenstr. 5, Postfach 1114, 40225 Düsseldorf, Deutschland; 2 Fakultät für Naturwissenschaften, Universität Genf, Genf, Schweiz

**Keywords:** Geschichte der Medizin, Volkmar Sigusch, Kastration, Sexuelle Orientierung, Geschlechtliche Identität, History of medicine, Volkmar Sigusch, Castration, Sexual orientation, Gender identity

## Abstract

Zu den neuen Forschungsfragen in der Geschichte der deutschsprachigen Sexualwissenschaft und Sexualmedizin zählen neben einem neuen Blick auf Kaiserreich und Weimarer Republik und Magnus Hirschfeld als Protagonisten dieser Zeit auch die Zeitgeschichte des Fachs in der Bundesrepublik mit den zwei prägenden Instituten in Frankfurt (Volkmar Sigusch) und Hamburg (Eberhard Schorsch). Auch in der Nachkriegszeit bestand die Tendenz weiter, gesellschaftliche Probleme durch endokrinologische und chirurgische Zugriffe lösen zu wollen. Hierzu gehört auch die (freiwillige) Kastration von Sexualstraftätern, die seit 1969 in der Bundesrepublik gesetzlich geregt ist. Nicht nur im Kontext von geschlechtsangleichenden Operationen stellen sich Fragen nach geschlechtlicher Identität. Diese haben auch hohe soziale Relevanz und werden in den letzten Jahren zunehmend politisiert. Diese Fragen sind auch anhaltend relevant für die Urologie und klinische Sexualmedizin.

## Abstract (en)

New research questions in the history of German sexology and sexual medicine include a new look at the Imperial and the Weimar Republic periods and Magnus Hirschfeld as a protagonist, as well as the contemporary history of the discipline in the Federal Republic with the two formative institutes in Frankfurt (Volkmar Sigusch) and Hamburg (Eberhard Schorsch). In the post-war period, the tendency to try to solve social problems through endocrinological and surgical approaches continued. This included the (voluntary) castration of sex offenders, which has been regulated by law in the West Germany since 1969. Questions of gender identity do not only arise in the context of gender reassignment surgery. They also have high social relevance and have become increasingly politicized in recent years. These questions are also persistently relevant for urology and clinical sexual medicine.

Die Leser:innen von *Die Urologie* haben in den letzten Jahren immer wieder Beiträge zur Geschichte der Sexualwissenschaft und Sexualmedizin des 19. und 20. Jahrhunderts v. a. im deutschsprachigen Raum in dieser Zeitschrift gefunden. Zuletzt waren dies etwa Beiträge zur Etablierung der Disziplin und Ergobiografien einzelner Fachvertreter [1–5]. Bereits ein Themenheft der Zeitschrift zum Schwerpunkt Sexualmedizin im Jahr 2006 war mit einer historischen Referenz eingeleitet worden [6]. In der Kultur- und der Geschichtswissenschaft ist das Interesse an der Fachgeschichte seit einigen Jahren enorm gestiegen. Dem Thema wird dabei zunehmend auch im Rahmen einer Zeitgeschichte der Sexualität begegnet [7, 8].

Das Terrain ist in der Tat noch kaum erschlossen. Bis vor wenigen Jahren war die historische Forschung im Wesentlichen auf die Geschichte der Sexualwissenschaft im Kaiserreich und der Weimarer Republik gerichtet gewesen. In besonderem Maße galt dies für das 1919 von Magnus Hirschfeld (1886–1935) in Berlin gegründete Institut für Sexualwissenschaft und die sexualpolitisch schon zuvor bedeutsamen Tätigkeiten seines Gründers [9, 10]. Dass auch jene, für die Disziplin schillernde, Epoche noch nicht als ausgeforscht gelten kann, demonstrierten jüngst mehrere Arbeiten. Sie haben sich etwa Fragen des fachhistorischen Wandels im und nach dem Ersten Weltkrieg gewidmet [11] oder Hirschfelds Positionen näher in den Kontext von Rassismus und Kolonialismus im ersten Drittel des 20. Jahrhunderts eingeordnet [12]. Von einer breiten Öffentlichkeit wahrgenommen wurde unlängst Rainer Herrns monumentale Geschichte des Hirschfeld-Instituts, welche die vielfältigen Tätigkeiten der Einrichtung auf der Höhe des historischen Forschungsstands kontextualisiert, sie v. a. mit Blick auf die Geschichte der Homosexuellenbewegung, des Sexualstrafrechts und sexuellen Aufklärungskonzepten einbettet [13]. Die Zerstörung des Instituts im Mai 1933 und das schon vor NS-Machtwechsel angesichts des allgegenwärtigen SA-Terrors bereits faktische Exil Hirschfelds, der sich 1930 auf eine Weltreise begab und 1935 im Exil in Nizza starb, bilden eine noch lange nachwirkende Zäsur in der Geschichte der deutschsprachigen Sexualwissenschaft, wobei die historische Forschung auch auf einige Kontinuitäten, die die NS-Zeit durchziehen, hingewiesen hat: Das gilt v. a. für die Rolle der Eugenik (Abb. 1).

**Figure 1:**
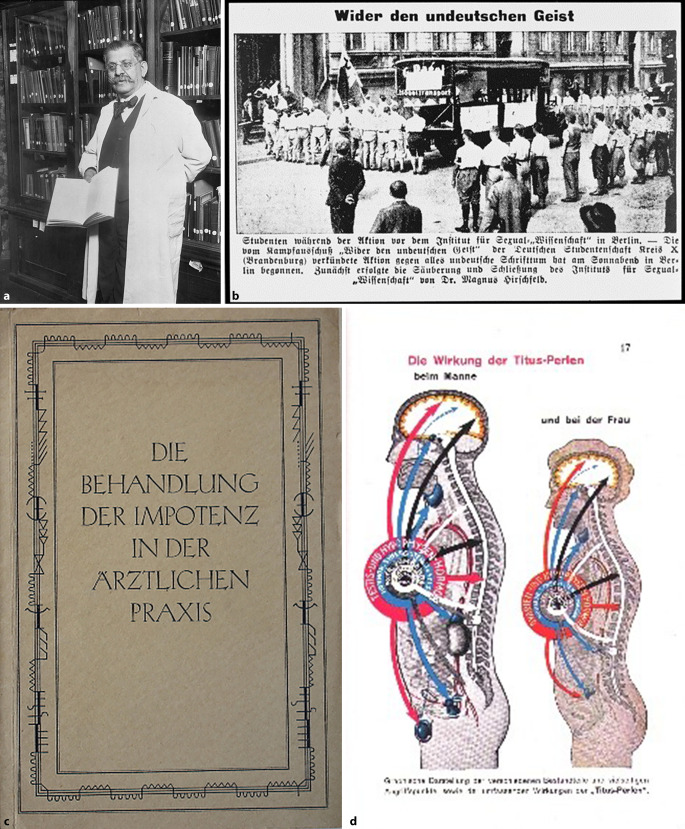

## Forschungsfragen

Wie sehr sich das historische Interesse inzwischen auch auf die Zeit nach 1945 ausgeweitet hat, wurde auf der im September 2022 in Erfurt abgehaltenen Tagung „Humanities“ der Gesellschaft für Geschichte der Wissenschaften, der Medizin und der Technik erneut deutlich. Die Gesellschaft widmete diesem Thema eine eigene Sektion. Wir dokumentieren im Folgenden in knapper Form Vorträge, die im Rahmen dieses von Matthis Krischel organisierten Tagungsabschnitts gehalten wurden. Zu diesem Zweck haben die Referierenden Richard Kühl (über die NS-Wissenschaftsgeschichte in der Perspektive der westdeutschen Sexualwissenschaft) und Matthis Krischel (über das bundesrepublikanische „Gesetz über die freiwillige Kastration und andere Behandlungsmethoden“ von 1969) Autoreferate ihrer jeweiligen Vorträge zur Verfügung gestellt, ebenso wie Dana Mahr (über biologischen Reduktionismus in Debatten zum Thema trans*), die nicht Teil der Sektion war.

Schlaglichtartig werfen diese Beiträge zugleich ein Licht auf einige Forschungstrends und neu aufkommende Fragestellungen, wie sie in der Sexualgeschichte generell diskutiert werden [14]. Neben medizin-, wissenschafts- und sozialhistorischen Fragekomplexen ergeben sich auch immer wieder Bezüge zur klinischen Sexualmedizin und Sexualwissenschaft im hier und jetzt.

## Richard Kühl: „Die Bewältigung der Vergangenheit ist gewaltsam“. Nationalsozialistische Wissenschaftsgeschichte in der Perspektive der westdeutschen Sexualwissenschaft der 1970er- und 1980er-Jahre

Deutsche Universitätskliniken haben sich nach 1945 ebenso wie medizinische Fachgesellschaften erst spät auf eine kritische Beschäftigung mit der eigenen NS-Geschichte eingelassen. Als frühe Gegenbeispiele gelten die großen Fachorganisationen der Kinderheilkunde und der Gynäkologie sowie einige wenige Medizinische Fakultäten, etwa diejenige in Freiburg im Breisgau. Wirkliche Durchbruchsjahre einer solchen Auseinandersetzungen bildeten jedoch erst die 2000er- und 2010er-Jahre [15]. Erst in diese Zeit fällt auch das von der Deutschen Gesellschaft für Urologie geförderte Forschungsprojekt zur Urologie im Nationalsozialismus [16, 17].

**Figure 2:**
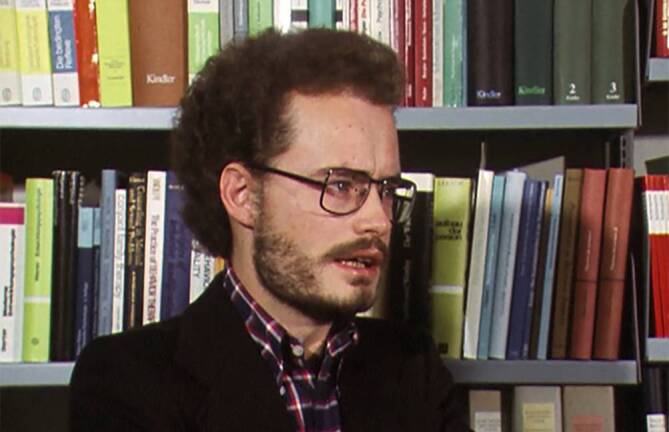

Weithin in Vergessenheit geriet in diesem Zusammenhang die enorme Präsenz historischer Bezüge auf die Wissenschaftsgeschichte des Nationalsozialismus innerhalb der westdeutschen Sexualwissenschaft um 1980. Diese Präsenz zeigte sich v. a. in der Publizistik der beiden damals wichtigsten universitären Fachinstitute in der Bundesrepublik, der Abteilung für Sexualwissenschaft der Universitätsklinik Frankfurt am Main (langjähriger Leiter: Volkmar Sigusch [1940–2023]; Abb. 2) und dem Institut für Sexualforschung und Forensische Psychiatrie der Universitätsklinik Hamburg-Eppendorf (Eberhard Schorsch [1935–1991]).

Anstoß dazu gaben am Ende der 1970er-Jahre kritische Stellungnahmen gegenüber Praktiken der Hirnchirurgie zur „Umpolung“ der sexuellen Orientierung sowie solche gegenüber endokrinologischen Forschungen zur „Ätiologie“ der Homosexualität und in den 1980er-Jahren schließlich auch die frühe Aids-Politik. Die Suche nach historischen Einordnungen zog eine Auseinandersetzung nicht nur mit der NS-Geschichte, sondern auch mit problematischen Traditionen der Sexualforschung der Zeit vor 1933 nach sich – v.a. auch mit ihren Bezügen zur Eugenik.

Wie unmittelbar in der eigenen Fachgeschichte das Humane und das Inhumane unter den ideellen und materiellen Bedingungen der Epoche der Moderne ineinandergreifen konnte, wurde um 1980 zur elementaren Grundeinsicht in den akademisch durchwirkten Sphären des Faches [18]. Dies führte nicht nur weg von apologetischen Lesarten einer wissenschaftshistorisch harten Zäsur der Jahre 1933 und 1945. Dieser doppelte Blick auf ideengeschichtliche Durchlässigkeiten der NS-Zeit nahm vielmehr Kernelemente des aktuellen theoretischen Rüstzeugs in der Wissenschaftsgeschichte vorweg. Bemerkenswerterweise fand dies *vor* den Debatten um Moderne und Ambivalenz statt, die gegen Ende der 1980er-Jahre neue Potenziale einer Historisierung der Moderne freilegten und deren destruktive Dimensionen erhellten [19].

Die Folgen dieser Auseinandersetzung innerhalb der Sexualwissenschaft waren vielfältig. Im Rückblick sind sie als bedeutender einzuschätzen als die „empirische Wende“, die gemeinhin zur Charakterisierung von Neuausrichtungen der bundesrepublikanischen Disziplinengeschichte in den 1970er-Jahren herangezogen wird. Für die Genese der noch bis in die 2000er-Jahre hinein in Deutschland einflussreichen „Kritischen Sexualwissenschaft“ waren sie wegbereitend [20]. Dieselbe Entwicklung wurde jedoch auch zum Anlass für innerfachliche Abspaltungen. Das fand Ende der 1970er-Jahre zunächst an der Peripherie der etablierten Zunft statt. Diese Segmente des Faches, die sich fortan als (vermeintlich) „unpolitisch“ definierten, gestalteten die Erweiterung der akademischen Infrastruktur des Faches in der „Berliner Republik“ indes in einem rückblickend erheblichen Maße. Die dramatische Verinselung der „Kritischen Sexualwissenschaft“ nach der Schließung des Frankfurter Instituts 2006 ist ohne diese im Kern geschichtspolitisch imprägnierte Vorgeschichte kaum zu verstehen.

## Matthis Krischel: Hormonforschung und chirurgische Herangehensweisen an soziale Problemkomplexe

**Figure 3:**
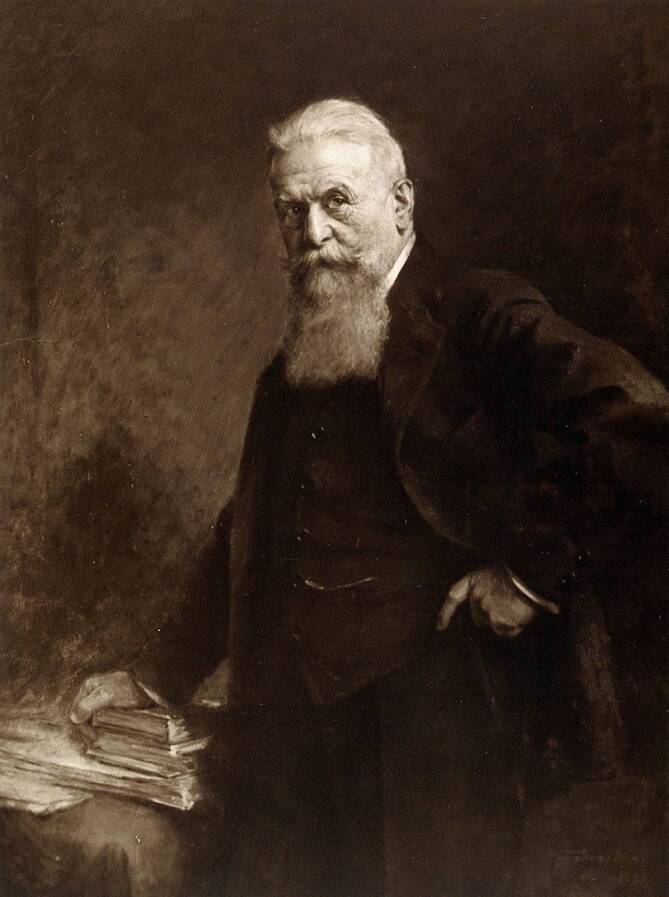

Zu den frühen Protagonisten der Hormonforschung zählte des österreichische Arzt Eugen Steinach (1861–1944 [21]; Abb. 3). Neben Vasektomien zur angeblichen Verjüngung propagierte er auch, dass männliche Homosexualität primär hormonell bedingt und durch chirurgische Transplantation von Hoden „heilbar“ sei [22]. Durch den SS-Arzt Carl Værnet (1893–1965) wurden mit diesem Ziel im Konzentrationslager Buchenwald an Häftlingen Versuche mit einer „künstlichen Drüse“ durchgeführt, welche implantiert wurde und Hormone abgab [23]. Jahrzehnte später verfolgte der ostdeutsche Endokrinologie Günter Dörner (1929–2018) ein Forschungsprogramm, in dem er danach fragte, ob durch Hormongabe an schwangere Frauen die Homosexualität ihres Nachwuchses verhindert werden könnte [24].

**Figure 4:**
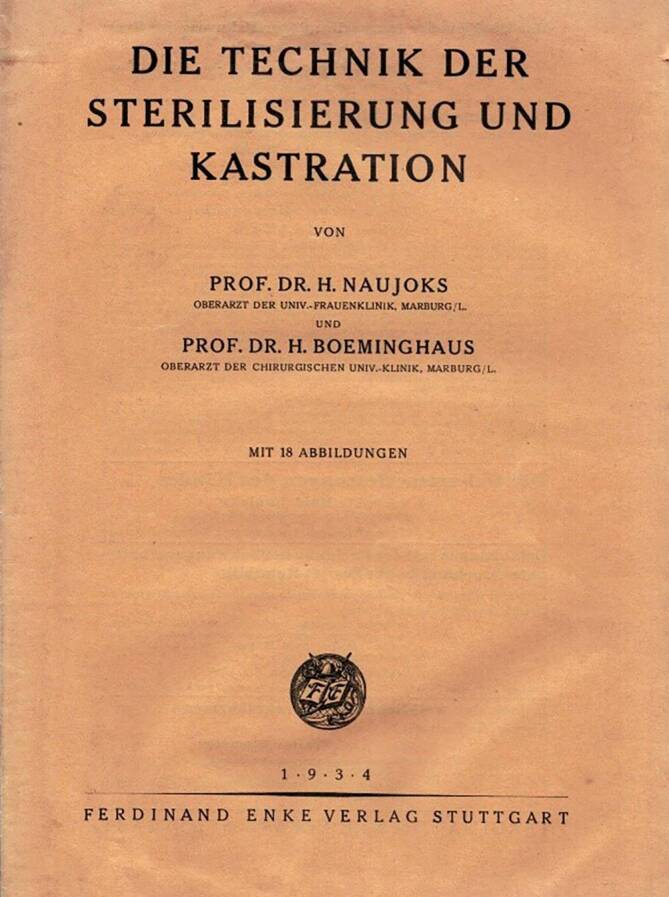

Der Ansatz, durch einen chirurgischen – die Orchiektomie – oder medikamentösen Eingriff den Hormonhaushalt von verurteilten Sexualstraftäter zu regulieren und so zumindest eine Triebdämpfung zu erreichen, kam und kommt in Deutschland bis heute zum Einsatz [25]. Mit dem bundesdeutschen „Gesetz über die freiwillige Kastration und andere Behandlungsmethoden“ ist eine entsprechende Regelung bis heute in Kraft, auch wenn die Anzahl der tatsächlich durchgeführten Operationen und chemischen Kastrationen in den letzten Jahrzehnten deutlich rückläufig ist. Die im Nationalsozialismus [26] durchgeführte, formal freiwillige, häufig durch Konzentrationslagerhaft und Folter erzwungene, Kastration (Abb. 4) von homosexuellen Männern mag im Nachkriegsdeutschland in Einzelfällen noch praktiziert worden sein, eine rechtliche Grundlage hatte sie jedoch nicht mehr [27]. Wie die forensische Kastration im Spannungsfeld zwischen Therapie und Strafe einzuordnen ist, wird bis heute in der Kriminologie diskutiert [28]. Bereits seit den 1930er-Jahren wird debattiert, ob von einer Freiwilligkeit gesprochen werden kann, wenn die Alternative zu einer Einwilligung „Schutzhaft“ oder – heute – die dauerhafte Unterbringung in der forensischen Psychiatrie ist [29].

Technische und Wissenskontinuitäten von der Kastration im forensischen Kontext zum therapeutischen Einsatz in der (Uro-)Onkologie stellen bisher noch eine Leerstelle der Forschung dar. Welche Rolle Hormone, innere und äußere Geschlechtsmerkmale sowie die soziale Rolle für Geschlechtlichkeit spielen, wird bis heute teils kontrovers diskutiert.

## Dana Mahr: Biologischer Reduktionismus und die Komplexität des menschlichen Geschlechts

„Was ist eine Frau?“ Mit dieser scheinbar einfachen Frage agitieren gegenwärtig konservative und rechte Akteur:innen gegen trans*-Menschen und ihre Rechte und fordern eine „eindeutige“ biologische Bestimmung von Geschlecht. Doch dieses Beharren auf „Biologie“ hat wenig mit Wissenschaft zu tun [30] und: die Diskussionen sind nicht neu [31].

Während andere wissenschaftsfeindliche Bewegungen der jüngeren Zeit im wesentlichen anti-faktisch argumentierten und als „Experten“ für ihr Anliegen höchstens akademisch randständige Figuren aufbringen konnten, berufen sich die Vertrer:innen des Biologismus des 21. Jahrhunderts auf ‚echte Wissenschaft‘ und haben tatsächlich akademisch hoch anerkannte Wissenschaftler:innen in ihren Reihen. Die Wissenschaft, auf die sich Akteure wie beispielsweise die Philosophin Kathleen Stock oder auch die Autorin J.K. Rowling (*1965) berufen, entspricht jedoch schon sehr lange nicht mehr dem Stand der aktuellen Forschung. Es handelt sich vielmehr um eine ideologisch motivierte reduktionistische Naturwissenschaftsnostalgie mit gesellschaftlichem und Hoheitsanspruch [32].

Dass derartige Denkfiguren nunmehr im deutschen Sprachraum angekommen sind, zeigt die verbissene Agitation gegen das von der Bundesregierung geplante Selbstbestimmungsgesetz. Mit diesem Gesetz soll das Leben für trans- und intergeschlechtliche Menschen verbessert werden. Insbesondere ist geplant, das bisherige Verfahren einer Personenstandsänderung zu vereinfachen. Wo früher ein langwieriges und kostenaufwendiges Gerichtsverfahren mit psychiatrischen Gutachten benötigt wurde, um im „Zielgeschlecht“ anerkannt zu werden, wird bald ein Verwaltungsakt genügen. Was für trans- und intergeschlechtlichen Menschen ein Grund zur Freude und Hoffnung ist, ist für andere ein Affront, ein Versuch, die Kategorie „Frau“ zu eliminieren, ja sogar ein Angriff auf die Biologie selbst.

Natürlich leugnen weder trans*-Menschen, intersexuelle Menschen noch Mediziner- oder Sexualwissenschaftler:innen die Binarität von Gameten oder die Existenz und Utilität der Kategorie „Frau“. Sie verweisen jedoch darauf, dass das menschliche Geschlecht jenseits solch einfacher Modelle und reduktionistischer Erklärungen hochgradig komplex ist. Der Verweis auf die geschlechtliche Vielfalt ist folglich kein Ausdruck der Leugnung der Biologie, wie von transfeindlichen Akteuren unterstellt wird, sondern ein wissenschaftlich gebotenes Zusammendenken verschiedener geschlechtsbestimmender Faktoren [33]. Die Berufung auf „nur die Gameten“ als Festlegung menschlicher Geschlechtszuordnung ist hingegen eine sowohl aus wissenschaftlicher als auch sozialer Perspektive Verkürzung längst vergangener Tage [34].

Anders als in Tiermodellen müssen beim Menschen die verschiedenen chemisch-biologischen Ebenen und die soziale Ebene zusammengedacht werden. Anders als es der politisierte biologische Reduktionismus rechter Akteure oder transfeindliche, selbsternannte „Feminist:innen“ behaupten, ist das biologische Geschlecht beim Menschen sehr komplex. Es ist nicht einfach eine Gegebenheit, sondern entsteht in komplexem Wechselspiel aus dem genetischen Geschlecht (1), dem morphologischen Geschlecht (u. a. Genitalien und Gameten) (2), der sexuellen Orientierung (3), der Geschlechtsidentität (4), sowie dem Ausdruck des eigenen Geschlechtsempfindens (5) einer Person, wie es beispielsweise der Neurowissenschaftler Steven Novella (*1964) prägnant dargelegt hat [35].

Die Anerkennung einer solchen Komplexität kann eine wichtige Bereicherung für die Diversifizierung medizinischer Praxis sein – auch und gerade in der Urologie, in der etwa der „klinische Blick der Sexualmedizin“ [36, 37] und geschlechtsangleichende Operationen eine Rolle spielen [38].

## Zusammenfassung

Die Urologie gehört bis heute zu den medizinischen Spezialisierungen, die zahlreiche Berührungspunkte mit der Sexualwissenschaft und -medizin aufweisen. Viele ihrer historischen Protagonist:innen arbeiteten im Bereich der Haut‑, Harn- und Geschlechtskrankheiten, beschäftigten sich aber auch mit der Erforschung der menschlichen Sexualität abseits von Krankheiten. Die hier vorgestellten Schlaglichter zeigen historische Forschungstrends auf, welche z. T. anschlussfähig an aktuelle sexualmedizinische und gesellschaftliche Fragen sind, darunter die nach dem Stellenwert und der Begründbarkeit der Kastration im forensischen Kontext sowie nach Geschlecht, Geschlechtlichkeit und geschlechtsangleichenden Operationen in Biologie, Medizin und Gesellschaft. Aber auch ganz konkrete, klinische Therapien wie das Verschreiben von Arzneimitteln zur Behandlung der erektilen Dysfunktion oder die Frage nach der Erhaltung der Potenz nach einer Entfernung der Prostata stellen solche Bezüge her.
